# Autoinflammatory Diseases and Physical Therapy

**DOI:** 10.31138/mjr.28.4.183

**Published:** 2017-12-22

**Authors:** Eda Gurcay, Aysen Akinci

**Affiliations:** 1 Gaziler Physical Medicine and Rehabilitation Education and Research Hospital, Ankara, Turkey,; 2 Hacettepe University Medical School, Department of Physical Medicine and Rehabilitation, Ankara, Turkey

**Keywords:** Autoinflammatory diseases, physical therapy, electrotherapy, exercise, rehabilitation

## Abstract

Autoinflammatory diseases (AIDs) constitute a group of clinical conditions, characterized by recurrent episodes of systemic inflammation, due to dysregulation of the innate immune system, without involving autoantibodies or antigen-specific T-cells. The patients exhibit recurrent episodes of fever with potentially serious complications and may have classic rheumatologic symptoms, including joint, skin, eye and muscle inflammation. Therefore, musculoskeletal problems and impaired quality of life can be anticipated as unavoidable consequences. In this regards any approach to ease the burden of symptoms and compensate the functional deficits are the main objectives of rehabilitation approach. For patients with inflammatory arthritis, physical therapy and rehabilitation methods have an important role in reducing joint pain and stiffness, preventing deformity, reconstructing muscle tissue and improving function. In order to justify the integration of rehabilitation approach in the management of AIDs and to determine the optimal protocols to use in this group of patients, well-designed, comprehensive, longitudinal, clinical trials using physical therapy centred outcomes are greatly needed.

## INTRODUCTION

Autoinflammatory diseases (AIDs) constitute a group of inherited disorders pathophysiologically characterized by unprovoked episodes of inflammation, due to dys-regulation of the innate immune system without antigen-specific T-cells and autoantibodies, in contrast to the autoimmune diseases. AIDs manifest clinically with recurrent episodes of fever, systemic inflammation in various organs which potentially are associated with serious complications and long-term disability. Additionally, the concept of autoinflammation has been extended to a number of other disorders of uncertain genetic aetiology including Behcet’s disease, Still’s disease, and Crohn’s disease (**Table 1**).^1–11^

**Table 1:** Genetic and clinical features of AIDs.

**AIDs**	**Inheritance**	**Duration of episode**	**MSKS involvement**	**Prominent features**	**Treatment**
***FMF***	AR	1–3 days	Monoarthritis, protracted in knees or hip	Periodic fevers (lasting 3–7 days), serositis, arthritis,	Colchicine IL-1 blockade in refractory cases
***Behcet’s disease***	Complex	Unpredictable pattern of exacerbation and remission	Arthralgia/arthritis-often in the knees/ankles	Recurrent oral/genital ulcers, erythema nodosum-like acneiform nodules, arthralgia/arthritis, eye/arterial/neurologic/GIS involvement	Steroids colchicine AZT, MTX, CyS IL-1 blockade
***SJIA***	Currently unknown	At least 2-week duration	Poli-/oligoarticular arthritis, most often in the wrists, knees, ankles	Remitting fever, erythematous skin rash, serositis, arthritis, LAP, HSM	IL-1 blockade IL-6 blockade
***AoSD***	Currently unknown	Fevers last for < 4-hr with rash, arthralgia	Arthralgia/arthritis are common	High spiking fever, arthralgia/arthritis, maculopapular rash, serositis, LAP, HSM	IL-1 blockade IL-6 blockade
***Crohn’s disease***	Complex	Remission and relapse	Migratory polyarthritis, sacroiliitis	Diarrhea, fever, arthritis, uveitis, skin rash, haematological/neurologic/cardio-pulmonary involvement	SLZ, steroids AZT, infliximab

*AIDs,* autoinflammatory diseases; *FMF,* familial Mediterranean fever; *SJIA*, Systemic Juvenile Idiopathic Arthritis; *AoSD,* Adult-onset Still’s Disease; *AR*, autosomal recessive; *MSKS*, musculoskeletal system; *GIS,* gastrointestinal system; *LAP*, lymphadenopathy; *HSM*, hepatosplenomegaly; IL, interleukin; *AZT,* azathioprine; *MTX,* methotrexate. *CyS*, cyclosporine; *SLZ,* sulphasalazine.

## METHODOLOGY

### Literature search and selection

A narrative review was performed using Medline/PubMed, Scopus and Google Scholar, with the keywords “autoinflammatory diseases”, “physical therapy”, “electrotherapy”, “exercise”, and “rehabilitation” which were selected from the medical subject headings (MeSH) terms to capture the most relevant papers on the topics in accordance with the literature. The search was limited to articles in the English language, starting from the earliest papers to May 2017 and carried out from the scientific search guidelines published in 2011.^12^

Since there are already published reports in the literature for inflammatory rheumatic diseases,^13–19^ we did not plan to address this issue in this article. Because the literature was scarce in AIDs, this overview contains general information and brief descriptions on some of the clinical entities of AIDs which are commonly encountered in the Physical and Rehabilitation Medicine (PRM) branch, particularly due to musculoskeletal involvement. The other issue to be addressed is physical therapy modalities and rehabilitation techniques. Therefore, the aim of our review was to highlight potential physical therapy and rehabilitation options for the most common AIDs in the context of the general principles of PRM blended with the use of inflammatory arthritis (**Table 2**).

**Table 2:** Physical therapy modalities and rehabilitative interventions used in inflammatory arthritis.

**Physical therapy modalities and rehabilitative interventions**	**Effects**
***Patient education***	Joint protection, energy conversation, coping with pain disability and the maintenance of work ability
***Rest and splinting*** *(acute stage)*	Reduce pain and inflammation, prevent joint stress/deformities
***Cold*** *(acute stage)* ***and hot*** *(chronic stage)* ***applications’***	Short-term relief of pain, joint stiffness, muscle spasm
***TENS*** *(acute and chronic stages)*	Reduce pain
***Hydrotherapy***	Increase ROM, strengthen muscle, relieve painful muscle spasm, improve the patient’s well-being.
***Assistive devices***	Reduce pain and functional deficits, keep patients’ independence and self-efficiency
***Exercise***	
*Strength and aerobic exercises*	Improve muscle strength, endurance, balance, aerobic capacity, and psychological well-being without exacerbating disease activity or joint destruction.
*Isometric exercise*	Adequate muscle tone
*ROM and flexibility exercise*	Increase the elasticity of periarticular tissues, prevent contractures

*TENS,* Transcutaneous electrical nerve stimulation; *ROM,* range of motion

## FAMILIAL MEDITERRANEAN FEVER

Familial Mediterranean Fever (FMF) is the most common of AIDs, transmitted as an autosomal recessive pattern, and is caused by mutations in the Mediterranean fever (MEFV) gene, which encodes the protein pyrin and is located on the short arm of the 16th chromosome. Although genetic transition has been found to cause FMF, environmental factors may increase susceptibility, and the exact pathogenesis of the disease remains unclear.^20–23^

Clinical manifestations appear early in the life in most of the patients. Although the MEFV is not mandatory for diagnosis, it has a 75% positive predictive value. The clinical findings include recurrent attacks of peritonitis, pleuritis, pericarditis, synovitis, febrile myalgia or erysipelas-like erythema associated with fever, resulting in pain in the abdomen, chest, joints, and muscles. The most serious complication is type AA amyloidosis, mainly affecting the kidney and is the cause of chronic renal failure.^21–24^

Musculoskeletal involvement in FMF occurs in 70%–75% of patients as acute attacks of mono- or oligoarthritis predominantly involving the large joints of the lower extremities with spontaneous resolution within 1 to 3 days; however, it may prolong up to 1 month or rarely longer, although less than 10% of FMF patients develop protracted arthritis and joint damage leading to disability, especially in the hips or knees.^25^ The presence of depression and the activity of serotonergic inflammatory cascade have been speculated to trigger FMF attacks.^26^ The inflammation in the knee joint is resolved without sequelae; in general, only joints with fluid aspiration. However, protracted hip arthritis may demonstrate destructive features.^27^ The incidence of sacroiliitis is increased in FMF patients, usually without the HLA-B27 antigen, while the pathogenic mechanisms that link these two conditions remain unknown.^28^ Besides, a rare case was represented by Keleş et al.^29^ as a co-existence of two conditions, ankylosing spondylitis (AS) and FMF, in a patient with a background of JIA.

Articular involvement is the initial symptom in one-third of patients and attacks can be precipitated by mild traumas, physical and emotional stress, exposure to cold, fat-rich meals, and infections. Patients with recurrent arthritis carry three-times higher risk for amyloidosis as compared to patients without articular involvement. Generally, attacks tend to decrease with aging.^24^ The attacks, that are often associated with increased levels of erythrocyte sedimentation rate, C-reactive protein, fibrinogen, and leukocytes, usually last for 1 to 3 days and resolve spontaneously; however, they may remain up to 1 month or rarely longer, and the interval between attacks is clinically relatively symptom-free.^30–31^ Colchicine is very efficacious in preventing FMF attacks and associated amyloidosis. Symptoms during attacks may be alleviated by non-steroidal anti-inflammatory drugs (NSAIDs), whereas usual dose of colchicine administration should be continued. Glucocorticoids may decrease the duration of attacks, but may also increase their frequency. Alternative biological treatments should be considered in cases that are resistant to, or intolerant of, colchicine.^24,32^ Certainly, the most significant goals should be to achieve appropriate treatment in order to prevent subclinical inflammation and the development of ‘silent’ amyloidosis, eventually to improve quality of life (QoL) in FMF as in other chronic diseases.

## BEHÇET’S DISEASE

Behçet’s disease (BD) is a chronic, inflammatory, and systemic disease affecting all types of blood vessels. Although vasculitis and perivascular inflammation are responsible for the pathology of the lesions, genetic predisposition, autoimmunity, and viral and bacterial infections are blamed for the etiology of BD; the origin of the disease still remains unclear.^33^

BD is found worldwide, but it is more common in a specific geographic area, defined as from Eastern Asia to the Mediterranean basin, compared with Western countries. The incidence of cases per 100,000 population varies from 80–370 in Turkey.^34^ The disease usually starts in the third decade of life.

BD is defined as a triple-symptom complex consisting of recurrent oral/genital ulcers and relapsing uveitis. In addition to the characteristic triad, common manifestations include skin lesions, articular, cardiovascular, pulmonary, neurological, and gastrointestinal systems involvement causing a variety of clinical problems which lead to functional disability.^35^ The arthritis of BD is generally mono- or polyarticular and usually follows an episodic and non-erosive pattern. In prospective studies, the incidence of arthritis ranges from 40 to 70%.^36,37^ The most common affected joints are knees, ankles, wrists, and elbows. However, it may infrequently masquerade as a destructive form of sacroiliitis or enthesitis, whereby differential diagnosis particularly from spondyloarthropathies may become challenging.^19^ BD lacks a universally reliable, specific laboratory test that projects the diagnosis and disease activity. Therefore, clinical findings become forefront for diagnosis. The symptoms demonstrate a chronic course with unpredictable pattern of exacerbation and remission.^38^

The disease can lead to increased mortality on account of large vessels, CNS, pulmonary and gastrointestinal systems involvement, particularly due to delayed diagnosis and treatment. In most patients with BD, arthritis can be managed with colchicine. IFNα, azathioprine and TNFα blockers may be tried in rare cases with resistant, longer lasting and disabling attacks. Therefore, the main target of treatment in BD is to prevent irreversible damage that mostly occurs early and active phase of the disease, and to prevent exacerbations of mucocutaneous and joint involvement, usually not causing damage but affecting QoL.^39,40^

## SYSTEMIC JUVENILE IDIOPATHIC ARTHRITIS AND ADULT-ONSET STILL’S DISEASE

Systemic-onset juvenile idiopathic arthritis (SJIA) is characterized by the presence of symmetric polyarthritis associated with intermittent high-grade fever (a typically daily high fever with spike in the evening) persisting for a minimum of 15 days with at least one of the following manifestations: skin rash (maculopapular or urticarial exanthema that accompanies fever), lymphadenopathy, hepatomegaly and/or splenomegaly, serositis in children and young adolescents <16 years of age. The clinical presentation of symptoms in SJIA is similar to that seen in adult-onset Still’s disease (AoSD) when it occurs in patients over the age of 16. Therefore, SJIA and AoSD likely represent a continuum of the the same disease entity.^41,42^ A distinctive feature of SJIA is its strong association with macrophage activation syndrome, characterised by an uncontrolled activation of macrophages releasing a high amount of pro inflammatory cytokines; particularly IL-1-related cytokines (IL-1β, IL-6, lL-18). The lack of any consistent association with HLA antigens or autoantibodies allow us to consider SJIA as an autoinflamma-tory disease.^43^ Finally, patients with SJIA, in contrast to patients with JIA, are at risk for amyloidosis, as in auto-inflammatory syndromes. The same considerations can be also made for AoSD, an uncommon clinical entity that predominantly affects young adults. In accordance with these findings is the dramatic and sustained efficacy of IL-1blockade on AoSD symptoms, even in refractory forms of the disease. Also, inhibitors of IL-6 were effective in controlling the activity of the disease.^44^ In order to improve clinical symptoms and QoL, to prevent long-term complications and to inhibit damage progression, the treatment of diseases should focus on controlling the inflammation. In conclusion, we can mention both SJIA and AoSD among the expanding group of AIDs.

## CROHN’S DISEASE

Crohn’s disease (CD) is a chronic, granulomatous inflammatory bowel disease that is believed to result from defects in both the adaptive and innate immune systems. The disease can affect the gastrointestinal tract at any point from the mouth to the rectum, but most commonly in terminal ileum, cecum, perianal area and colon. The symptoms of CD include abdominal pain, diarrhoea, fatigue, fever, gastrointestinal bleeding, and weight loss. Extraintestinal manifestations may include erythema nodosum, pyoderma gangrenosum, uveitis, spondylitis, sacroiliitis, peripheral arthropathy, hepatobiliary complications, genitourinary and renal manifestations hematological disorders, cardio-pulmonary manifestations, vasculitis, and neurological involvement.^45^ Patients with CD frequently present in adolescence with the median age of 20 to 30 years. Therapeutic recommendations are determined by disease location, activity, and associated complications. The main goals of treatment are to control inflammation, to achieve clinical remission with minimal adverse effects and to permit life to be lived as normally as possible. Treatment usually consists of anti-inflammatory agents, antibiotics, immunomodulators and disease-modifying agents.^46^

## PHYSICAL THERAPY

AIDs, including FMF, BD, SJIA, AoSD, or other AIDs contribute to the difficulties of life standard and its maintenance and can further reduce QoL, mainly due to physical symptoms associated with disease activity such as arthritis.^47–49^ The manifestations of AIDs, such as chronic joint pain, stiffness and deformity as well as adverse events due to pharmacological treatment or the disease itself may lead to cardiovascular deconditioning, muscle weakness, and poor endurance. Patients are often prone to rest due to pain, impaired exercise capacity and muscle strength. Pain frequency and intensity are higher in JIA than in other rheumatic diseases.^49^ Similarly, arthritis in BD affects the patients’ pain levels and may lead to temporary or permanent functional disabilities.^48^

Physical therapy and rehabilitation have important effects in relieving pain and stiffness, preventing deformity, rebuilding muscle tissue, developing dexterity, and improving function. Actually, physical therapy modalities including thermotherapy, electrotherapy, and balneotherapy frequently reduces the need for pharmacologic therapies.^50–52^

### Patient education

Patient education is of outmost importance for the individuals to manage the consequences of the diseases. Educational interventions include energy conservation/fatigue management, sleep hygiene training, pain relief strategies, relaxation training, and exercise recommendations which have significant effects on functional disability and psychological status.^51–53^

### Assistive devices

Despite optimum pain control and exercise therapy, full function cannot be restored. In such cases, function may be improved by the use of proper assistive devices for daily living activities and transfer. In patients with BD who have CNS involvement may end with upper motor neuron or cerebellar impairment that can interfere with functioning.^54^ Assistive devices such as canes, crutches, walkers, ankle-foot orthoses, or knee braces may be important for the realization of highest ambulatory potential.^55,56^

### Thermotherapy

Thermotherapy includes a cold or a hot source or a contrast bath with water submersions. Local application of a cold (ice packs, ice massage, or sprays) or a hot (hot packs, hot moist towels, warm water, paraffin baths, or infrared radiation), and deep heat, (shortwave diathermy or ultrasound) source can raise the pain threshold and decrease joint stiffness.^57^ A basic rule is that chronic pain or inflammation seems to respond better to heat and acute pain better to cold. The usual duration of application of heat or cold is 20 to 30 minutes daily, and most can be prescribed for home use.^50^ Contrast baths decrease inflammation, swelling, pain, and joint stiffness. While a systematic review on the effectiveness of contrast baths indicated no physiologic effect on intramuscular temperature and the lymphatic system, an increase in skin temperature and superficial blood flow down to the subcutaneous level was noted.^58,59^ Paraffin baths and infrared radiation combined with exercises can be recommended for beneficial short-term effects for arthritic hands.^57^ The limitations of thermal therapy include incomplete alleviation of symptoms and the inability of some patients (particularly those with circulatory or sensory deficits) to tolerate a sufficiently intense thermal stimulus. It should be used as an adjunctive method of pain control and/or in preparation for exercise therapy.^50^

### Hydrotherapy

Hydrotherapy provides a combination of buoyancy and superficial heating for the body or body parts by immersion in a pool or a Hubbard tank containing warm water. The warmth and antigravity effects of hydrotherapy can facilitate exercise and ambulation, particularly for those with severe large-joint involvement.^56,63^ The exercises contain aerobic, resistance, and stretching activities in water, providing similar physical benefits to exercise on land but may have additional and important psychological effects.^60,61^

Balneotherapy is the medical practice of treatment by immersion in baths, filled with thermal water with a significant mineral content. Different types of mineral water such as salt or mineral baths, sulphur baths, and radon-carbon dioxide baths may be used in this therapy.^59,62^ The mineral water reaction includes tiredness and fatigue especially after 5–8 baths with an associated rise in the leukocyte count and erythrocyte sedimentation rate, even within the normal range.^63^ The aims of balneo-therapy are to relieve pain and improve joint motion and, consequently, to make the patients feel well. Yurtkuran et al*.*^64^ conducted a clinical trial comparing spa therapy to NSAIDs and a combination of both in AS patients and found that spa therapy was more effective in relieving symptoms and improving spine mobility than NSAIDs alone, with the effect lasting up to six months. Falagas et al.^65^ reported a review of balneotherapy in rheumatic diseases and found a possibility that balneotherapy is associated with clinical improvement in osteoarthritis, fibromyalgia, AS, rheumatoid arthritis and chronic low back pain.

### Electrical therapy

*Transcutaneous electrical nerve stimulation* (TENS) has been widely used as a treatment modality for acute and chronic pain, and is reported to relieve pain in 20–40% of patients with chronic, non-malignant pain and 70–90% of patients with postoperative pain.^66^ It has occasionally been proven beneficial in chronic arthritic knee joint pain that was unresponsive to other kind of therapies. Mannheimer and associates found that high-intensity TENS reduced joint pain in patients with RA.^67^ The theoretical mechanism of action of TENS is thought to involve inhibition of pain impulse transmission at the level of the substantia gelatinosa of the spinal cord and alteration of pain perception more centrally. The other possible mechanism is the release of endogenous opioids.^68,69^ Initially, TENS is carried out in the PRM departments, and if it is effective then instructions are given for home use due to its simple and safe application.^50^

*Interferential current* is an electrotherapeutic technique which is able to penetrate deeply within the areas of treatment, predominantly used for treatment of pain. Therefore, it could be used for managing chronic painful conditions including in patients with JIA.^49,70^

*Low-level laser therapy* (LLLT) is another modality to relieve pain and to improve function in patients with musculoskeletal involvement. The laser emits a single wavelength of pure light, which causes a photochemical reaction within the cell. LLLT decreases pain and morning stiffness for up to four weeks in people with rheumatoid arthritis, meaning that the effect is not long-lasting.^59,62^

*Therapeutic ultrasound* (US) provides high frequency mechanical vibrations in a continuous fashion to relieve pain by its thermal effects or with pulsed sequencing to reduce inflammation. The heating effects of continuous ultrasound can also reduce muscle spasms and stimulate blood flow to decrease inflammatory toxins. It can penetrate deeper tissues, such as collagen, to increase its elasticity. US applied to the dorsal and palmar aspects of the hand showed increased grip strength compared to the placebo.^59,62,71^

### Therapeutic exercise

Therapeutic exercise commonly provokes a favourable response in terms of physical and psychological benefits in patients with arthritis. Exercise therapy on land and in water covers sports and recreational/occupational/daily-living/aerobic activities, flexibility and muscle-strengthening/core stability exercise, and balance rehabilitation for modifying health status. It consists of planned, structured and repetitive movement of parts or the whole body.^50,59,72^

Arthritic involvement of a joint always causes loss of strength in the associated muscle groups; usually at a rate of 30% per week.^50^ Children and adolescents with JIA have decreased muscle strength when compared with their normal peers. Therefore, physiotherapy and occupational therapy are important components of the rehabilitation approach to patients with JIA. Exercises which do not give weight to joints, especially swimming and cycling, should be encouraged, especially in the early stages.^73,74^

The prescription of an exercise program should be as attentive as a medical prescription. Therefore, in the exercise prescription, pain threshold, exercise physiology, patient’s functional limitations, and disease activity must be taken into account. During the exacerbations, the exercises should be performed in inpatient or an outpatient clinic under supervision. It is not always easy to answer the questions about how often, in what order, and when which exercises should be introduced: exercises should be performed when the patient feels best. As pain control is usually necessary before exercise, this may be needed also after exercise.^50,52,59^

The purpose of therapeutic stretching exercises is to prevent contracture or to increase range of motion with passive, active assisted and active exercises. Passive stretching has little place in arthritis therapy. Active-assisted stretching is used when the patient can initiate an exercise in order to achieve the joint’s available range of motion. Active stretching is generally preferred in the protection of range of motion. A stretching exercise program should be designed with the least stressful movements. The purpose of therapeutic strengthening exercises is to develop strength and endurance in specific muscle groups to facilitate functionality. The two major categories of strength are isometric and isotonic. Isometric (static) strength may be defined as the maximum force that can be applied against immovable objects by a muscle group. It is an optimum treatment for weakness associated with arthritis. Isotonic (dynamic) strength is the maximum force that can be performed in moving an object.^50–59^

Aerobic (dynamic) exercise programmes improve the components of health-related fitness, enhance psychological status, reduce pain and fatigue and have a positive effect on functional capacity without exacerbating disease activity or accelerating joint damage.^50–59^

Specific exercises are an effective adjuvant therapy to enhance cardiopulmonary functions in patients with AS. In addition to the medical treatments, specific exercise therapy might reduce the cardiopulmonary complications related with AS.^18^ While moderate evidence supports exercise interventions in improving physical function, disease activity and chest expansion compared to controls; there is low-level evidence of improved pain, stiffness, spinal mobility and cardiorespiratory function. Supervised group exercise provides more beneficial outcomes than unsupervised home exercise.^19^ Geneen et al.^14^ suggested physical activity and exercise in pain severity reduction and improved physical function for adults with chronic pain; though these were mostly of small-to-moderate effect, and were not consistent across the reviews.

### Limitations

The main limitation of this report is that it is structured a narrative review. While narrative reviews can provide experts’ intuitive and experiential perspectives on focused topics, systematic reviews are based on the findings of comprehensive and systematic literature searches in all available resources, with minimization of selection bias. Additionally, we did not specifically search non-English language databases. Thus, we may have failed to identify potentially relevant reports that have been developed in a non-English language setting. A further limitation was that evidence supporting the use of specific physical therapies in most AIDs is lacking. Thus, in this report we recommend some therapeutic options in the context of the general principles of PRM in **Table 2**.

## CONCLUSION

In conclusion, patients with AIDs have a disturbed musculoskeletal system and impaired QoL. To ease the burden of symptoms and compensation of functional deficits are the main objectives of rehabilitation approach. Although rehabilitation may not always have a direct influence on the progression of a disease, it enhances personal activities and participation in social activities, thereby improving QoL. While literature review on physical therapy modalities and rehabilitation interventions demonstrates beneficial effects on different symptoms at various levels in inflammatory arthritis, evidence in AIDs is still scarce. In order to justify the integration of rehabilitation approach in the management of AIDs, well-designed, comprehensive, longitudinal, clinical trials using physical therapy centred outcomes are greatly needed.

## SUMMARY OF KEY POINTS

- AIDs are a group of genetically different but clinically similar disorders unified by recurrent episodes of fever and systemic inflammation, with potentially serious complications.
- Exercise is the most studied physiotherapy modality in terms of pain, function, and QoL for inflammatory arthritis, with few studies examining other physiotherapy modalities.
- Future work is needed in AIDs to clearly establish the role of physical therapy modalities and to determine the optimal protocol to use in this group of patients.

## DISCLAIMER

The authors state that no part of the text of tables was copied from elsewhere, and all ideas presented in the manuscript are those of the authors.
